# A comprehensive transcriptome and immune-gene repertoire of the lepidopteran model host *Galleria mellonella*

**DOI:** 10.1186/1471-2164-12-308

**Published:** 2011-06-11

**Authors:** Heiko Vogel, Boran Altincicek, Gernot Glöckner, Andreas Vilcinskas

**Affiliations:** 1 Max Planck Institute for Chemical Ecology, Hans-Knoell-Strasse 8, 07745 Jena, Germany; 2 INRES-Phytomedicine, Rheinische Friedrich-Wilhelms-University of Bonn, Germany; 3 Leibniz-Institute of Freshwater Ecology and Inland Fisheries, IGB Müggelseedamm 310, D-12587 Berlin, Germany; 4 Institute of Phytopathology and Applied Zoology, University of Giessen, Heinrich-Buff-Ring 26-32, 35392 Giessen, Germany

## Abstract

**Background:**

The larvae of the greater wax moth *Galleria mellonella *are increasingly used (i) as mini-hosts to study pathogenesis and virulence factors of prominent bacterial and fungal human pathogens, (ii) as a whole-animal high throughput infection system for testing pathogen mutant libraries, and (iii) as a reliable host model to evaluate the efficacy of antibiotics against human pathogens. In order to compensate for the lack of genomic information in *Galleria*, we subjected the transcriptome of different developmental stages and immune-challenged larvae to next generation sequencing.

**Results:**

We performed a *Galleria *transcriptome characterization on the Roche 454-FLX platform combined with traditional Sanger sequencing to obtain a comprehensive transcriptome. To maximize sequence diversity, we pooled RNA extracted from different developmental stages, larval tissues including hemocytes, and from immune-challenged larvae and normalized the cDNA pool. We generated a total of 789,105 pyrosequencing and 12,032 high-quality Sanger EST sequences which clustered into 18,690 contigs with an average length of 1,132 bases. Approximately 40% of the ESTs were significantly similar (*E *≤ e^-03^) to proteins of other insects, of which 45% have a reported function. We identified a large number of genes encoding proteins with established functions in immunity related sensing of microbial signatures and signaling, as well as effector molecules such as antimicrobial peptides and inhibitors of microbial proteinases. In addition, we found genes known as mediators of melanization or contributing to stress responses. Using the transcriptomic data, we identified hemolymph peptides and proteins induced upon immune challenge by 2D-gelelectrophoresis combined with mass spectrometric analysis.

**Conclusion:**

Here, we have developed extensive transcriptomic resources for *Galleria*. The data obtained is rich in gene transcripts related to immunity, expanding remarkably our knowledge about immune and stress-inducible genes in *Galleria *and providing the complete sequences of genes whose primary structure have only partially been characterized using proteomic methods. The generated data provide for the first time access to the genetic architecture of immunity in this model host, allowing us to elucidate the molecular mechanisms underlying pathogen and parasite response and detailed analyses of both its immune responses against human pathogens, and its coevolution with entomopathogens.

## Background

The introduction of novel high through-put sequencing technologies provides insight into the genetic architecture of an increasing number of non-model organisms including insects. Next-generation (NextGen) pyrosequencing has become an important tool in transcriptomic studies and allows targeted identification of genes which are (differentially) expressed in distinct tissues or cells, during development, or upon activation of immune responses. This technology has been used, for example, to characterize both the midgut-specific and the immunity-related transcriptome of *Manduca sexta*, which has emerged as a model in lepidopteran biochemistry and physiology [1,2]. In this study, we subjected the immunity-related transcriptome of the greater wax moth *Galleria mellonella *to a combination of Sanger and NextGen sequence analysis. Our study was motivated by two reasons. Firstly, Galleria is suited to identify ancient features of innate immunity in lepidopterans because it belongs to the family Pyralidae which has been placed in a basal phylogenetic position within the Lepidoptera. Secondly, *Galleria *represents a powerful, reliable and proven model system for innate immunity studies. It is currently used as a host system to reconstruct rapid reciprocal adaptations during host-parasite coevolution [3] and as a an alternative model host for testing human pathogens, which is ethically better acceptable than mammalian hosts such as mice, rats and rabbits [4,5]. *Galleria *caterpillars prosper world-wide in use as alternative mini-hosts because they combine advantages shared with other invertebrate host models with benefits that are unique to this lepidopteran. The advantages of the nematode *Caenorhabditis elegans *and the fruit fly *Drosophila melanogaster *are complete, well-annotated genomes and that microarrays, RNA interference libraries and mutant strains are available which allow analysis of host-pathogen interactions at the molecular level [6]. However, the larger size of *Galleria *caterpillars enables precise injection of antibiotics or a number of pathogens, easy manipulation and collection of tissue and hemolymph samples to study pathophysiology with, for example, proteomic approaches.

Further advantages of *Galleria *are (i) the low overall costs of breeding large numbers, providing an inexpensive whole-animal high throughput infection assay system [7], (ii) their worldwide commercial availability, e.g., they are sold as bait for fishermen or as food for pets (reptiles), (iii) the positive correlation between the pathogenicity of bacteria and fungi when evaluated in *Galleria *and mice [8], (iiii) and that this heterologous insect host can be adapted in the laboratory to human physiological temperature (37°C). This is essential in order to mimic the physiological conditions in mammals because human pathogens are adapted to the physiological temperature of their host which is often required for the synthesis and the release of their pathogenic or virulence factors [4,5]. These advantages have convinced an increasing number of researchers to favor *Galleria *as a mini-host model for prominent pathogenic bacteria and fungi that are responsible for severe human diseases such as *Bacillus cereus *[9], *Enterococcus faecalis *[10], *Listeria monocytogenes *[11]*, Pseudomonas aeruginosa *[12], *Staphylococcus aureus *[13], *Candida albicans *[14] and *Cryptococcus neoformans *[15]. In addition, a number of antimicrobial peptides and inhibitors of microbial virulence factors have been discovered during the past decade in *Galleria *whose therapeutic potential in medicine and plant protection is presently being explored [16,17].

The major disadvantage of *Galleria *as a heterologous host system is that neither genome nor transcriptome sequence data are available and, therefore, important information about the immunity and stress related genes and their expression are lacking. Consequently, this study was designed to fill this gap and to provide a data set which enables more detailed studies, for example microarray or proteomic analysis, in the future. In order to induce expression of immunity-related genes in this lepidopteran species we injected a bacterial lipopolysaccharide (LPS) preparation into last instar larvae which has been proven as a potent elicitor of immune responses in *Galleria *[18] and other insect species [19,20]. Normalized larval dscDNA was sequenced using Roche 454 FLX and Sanger (directional long reads) methods. The combining of both technologies provided deep sequencing coverage of the expressed genes relevant to this research project.

Because of the large hemolymph sample volumes that can be obtained from *Galleria *caterpillars, their host response to pathogens can easily be studied at the peptide and protein level [21]. To test the correlation between transcriptomic and proteomic data we collected hemolymph samples from untreated and LPS-injected larvae. In order to identify peptides and proteins that are secreted within the hemolymph upon activation of innate immune responses, we used 2D-gelelectrophoresis combined with mass spectrometric analysis of spots that appear or are enhanced upon injection of LPS. Complementary proteomic analysis of hemolymph samples confirmed induced expression and release into the hemolymph of proteins known to mediate recognition of microbes, immunity-related signaling or killing of microbes.

## Results and Discussion

### Transcriptome assembly and functional analyses using Gene Ontologies

*Galleria *has emerged as a powerful, surrogate and ethically acceptable model host for human pathogens, but the lack of genomic data is a major impediment for its use in preclinical research. In order to provide more detailed information about its transcriptome we subjected normalized larval cDNA to NextGen pyrosequence (Roche 454 FLX) and Sanger analysis. Normalization of the larval cDNA resulted in reduction of any over-abundant transcripts and production of an even distribution of transcripts ranging from 0.2 to > 3.0 kb in size. The average size of the cDNAs of the *Galleria *mixed larval cDNA library that were cloned and sequenced was 1,150 bp. With the 454 platform we generated over 172 million bases sequence information. The total number of reads was 789,105 with an average length of read (bases) of 218. The data assembly consisted of 22,203 contigs and 11,7 million bases among which 7,265 contigs were larger than 500 bases. The average contig size of contigs larger than 500 bases was 1,132 bases. The largest contig had 4,762 bases, the N50 contig size was 1,309 bases. The Sanger sequencing yielded a total of 12,032 high-quality ESTs, which after assembly resulted in a total of 2,120 contiguous sequences (contigs) and 4,775 singletons represented by a single EST and a total of 6,895 putative gene objects (summarized in table 1). Combined, the Sanger and the pyrosequencing data of the *Galleria *larval cDNA library resulted in a total of 18,690 contigs (putative gene objects). For the subsequent BLAST searches and annotation, we excluded all of the singleton reads from the 454 pyrosequencing data as the singlets may be more error-prone and unreliable as compared to contigs with good sequence coverage. The complete set of sequences were subjected to a protein translated BLASTx search and a gene ontology (GO) analysis using Blast2GO [22]. 7,556 Sequences (40%) matched described sequences in Genbank (NR database; E-value cut-off of 10^-3^). More than half of the sequences (11,134) had no BLAST result, indicating a high number of lepidopteran or species-specific transcripts or transcript parts (orphan UTRs) [23].

**Table 1:** Summary statistics for *Galleria mellonella *expressed sequence tag (EST) analysis

	Sanger technology	454 FLX technology	Sanger + 454 FLX
Total number of reads	14,120	789,105	**-**
Average length of read (bases)	780	218	**-**
Total number of reads in the assembly	12,032	466,737	478,769
Total number of contigs	2,120	22,203	18,690
Total number of singlets	4,775	25,604	**-**
Number of unique sequences	6,895	47,807	**-**
Average/Largest contig size	995/3,560	1132/4,762	1180/4,762

**Figure 1:**
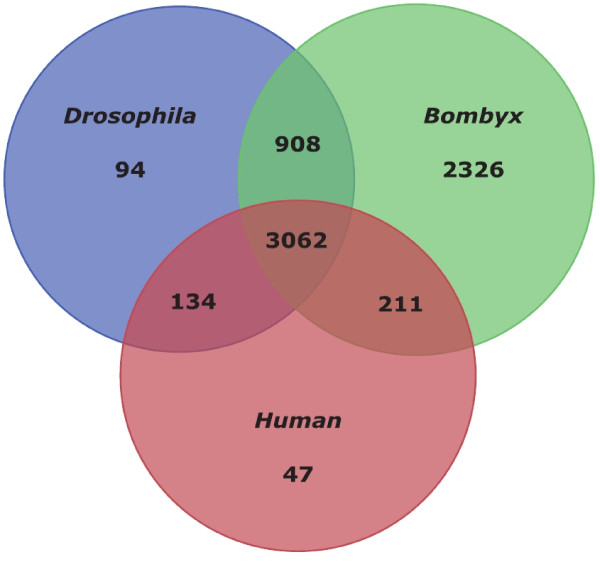
**Orthologous genes shared between *Galleria, Bombyx, Drosophila *and humans**. The Venn diagram shows the number of orthologous groups of genes shared between *Galleria, Bombyx, Drosophila*, and human genomes/transcriptomes. A larger fraction of the *Galleria *transcripts is shared with all species but the majority of *Galleria *genes are shared with *Bombyx mori *only.

We then analyzed which part of the assembled contigs had counterparts in certain species. For this purpose we used BLAST databases for the complete proteomes of *Drosophila melanogaster, Bombyx mori*, and *Homo sapiens*. Using this approach we cannot discern gene family members but get a rough overview on general protein distributions between clades. The Venn diagram (Figure 1) shows the distribution of hits with a score threshold of 150. We chose the score value as excluding criteria to make blast hits comparable, since p values are influenced by the data base size. We observed a total of 6782 hits of which more than 3000 are common to all species and therefore belong to the core of all metazoan genes. Given that not all genes are active under the conditions chosen and that there are a number of species specific genes, the 6507 identifiable genes shared between *Bombyx *and *Galleria *indicate a good coverage of the *Galleria *transcriptome by our contig data set. Not surprisingly another large fraction (2326) is shared between the Lepidoptera only and 908 contigs are common between Diptera and Lepidoptera. The slightly higher number of genes shared between Lepidoptera and humans than between *D. melanogaster *and humans (211 versus 134) indicate a *D. melanogaster *specific gene loss. Overall, the species distribution of the top BLAST hit against the nr database for the *Galleria *transcriptome shows a strong preference for matches against *Bombyx mori *and *Tribolium castaneum *genes. Both insect species are represented by complete genome sequences in the public databases, as for *Drosophila*, but the number of top BLAST hits against this insect model organism is much smaller (Additional file 1).

**Figure 2:**
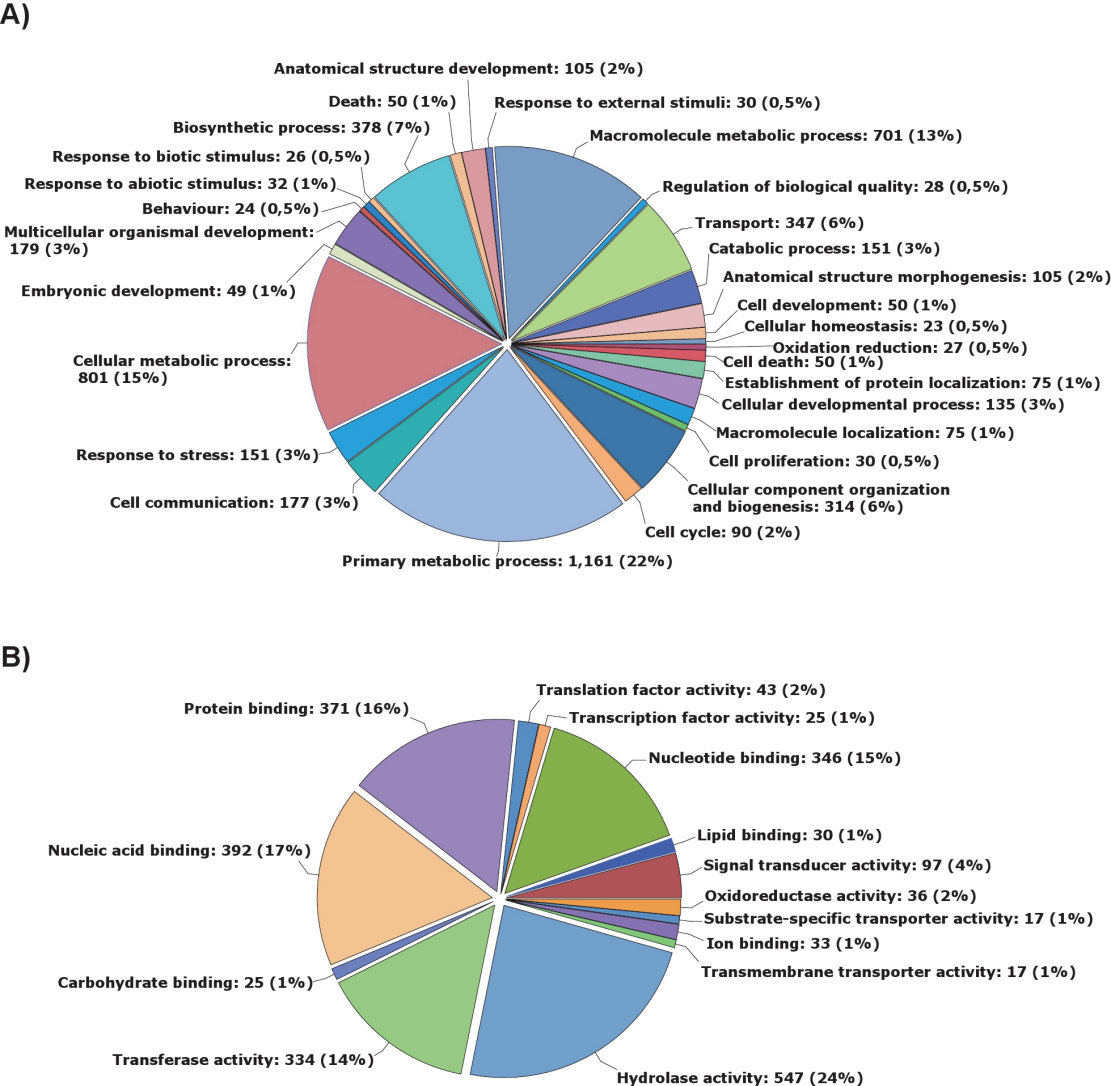
**Gene ontology (GO) assignments for the *Galleria *transcriptome**. GO assignments as predicted for their involvement in (A) biological processes and (B) molecular functions. All data are presented at level 3 GO categorization. Classified gene objects are depicted as absolute numbers and percentages (in brackets) of the total number of gene objects with GO assignments.

For functional comparisons, all sequences were subjected to Gene Ontology (GO) analysis in Blast2GO, where we classified all gene objects in Biological Function (Figure 2A) and Molecular Process class 3 (Figure 2B). To minimize the number of classes with only few gene objects, we set the minimum number of gene objects (cut-off level) in a class to 2% of the total number of sequences that could be classified. Of the 7,556 contigs in the *Galleria *cDNA library with high-score matches in the Genbank non-redundant (nr) protein database, 3,438 (45%) could be classified into a GO category, with each class containing at least 17 sequences (2% of 3,438). Among the 3,438 genes for which we obtained GO terms, we observed a wide diversity of functional categories represented on all levels of the Gene Ontology database. Figure 2 shows a total of 28 (Biological Process) and 14 (Molecular Function) GO level-3 classes into which the gene objects were classified. The most prominent GO Biological Process categories were Cellular metabolic process, Macromolecule metabolic process and Primary metabolic process (each with 13-22% of the total), followed by the classes Biosynthetic process, Cellular component organization and biogenesis, and Transport (each with about 6-7%). The most prominent GO Molecular Function categories were Protein Binding, Nucleic Acid Binding and Hydrolase Activity (each with 16-24% of the total hits). A comparison of the GO categorized *Galleria *transcripts with a functional GO category and the complete predicted Unigene set from the silk moth (*Bombyx mori*) genome subjected to the same Blast2GO process revealed a substantial overlap between the two datasets. Out of 16,425 total *Bombyx *Unigenes, 12,076 had a significant BLAST hit (NR database; E-value cut-off of 10-3), a much larger fraction (73%) as compared to the *Galleria *sequence data set (40%). Of the 12,076 genes with a BLAST hit 6,058 *Bombyx *genes had a functional GO category association (Additional file 2). In a direct comparison of the presence and/or abundance of GO terms, three GO classes were absent in the *Galleria *transcriptome, but present in the *Bombyx *Unigene dataset: Generation of precursor metabolites and energy, Cell recognition and Regulation of biological process. Based on the relative numbers of gene objects with functional GO category associations the *Galleria *sequences fall into GO categories with a roughly similar distribution to that of the *Bombyx *genome showing comparable numbers of most GO categories both for Biological Process and Molecular Function (Additional file 2). This suggests that the *Galleria *sequence data contain a large diversity of genes involved in a variety of biological processes, and do not contain notable biases towards particular categories of genes.

The patterns of GO category associations nonetheless differed between these two insect species in a few categories, with relatively high abundance of Multicellular organismal development, Anatomical structure morphogenesis and Cellular developmental process in *Bombyx *and Biosynthetic process and Macromolecule metabolic process and response to stress being more abundant in Galleria. In addition to this, several categories were only present in *Galleria *(Oxidation reduction and Cell death). Differences in GO category associations between *Bombyx *and *Galleria *might be attributed to the fact that ESTs of the latter originate predominantly from larvae.

### Recognition of pathogen or damage associated molecular pattern genes

**Figure 3:**
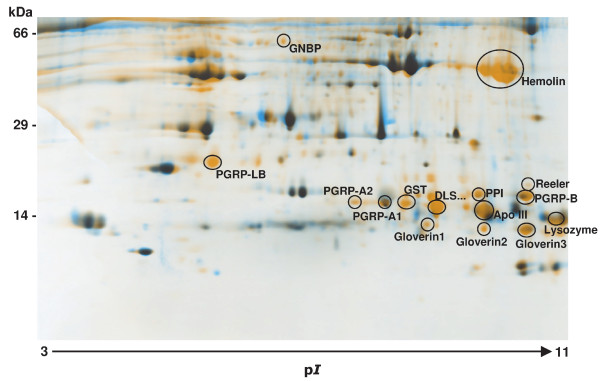
**Two-dimensional SDS-Page map of immunized *Galleria *larvae**. Hemolymph protein from untreated and LPS-immunized larvae was loaded on 24-cm pH 3 to 11 NL isoelectric focusing strips, followed by Tris-Tricine-SDS-polyacrylamide gel electrophoresis on a 15% gel. Image analysis enabled visualization of new or enhanced spots present in hemolymph samples from immunized larvae depicted in orange color. Putative identifications of immune-inducible proteins by MALDI-TOF analysis and according to our recent study [21] are depicted next to the respective spots. Molecular mass standards are indicated in kDa (left), and the pI range by an arrow. PGRP, peptidoglycan recognition protein; GNBP, Gram negative bacteria binding protein; GST, Glutathione-S-transferase; Apo III, apolipophorin III; DLS..., unknown protein.

**Figure 4:**
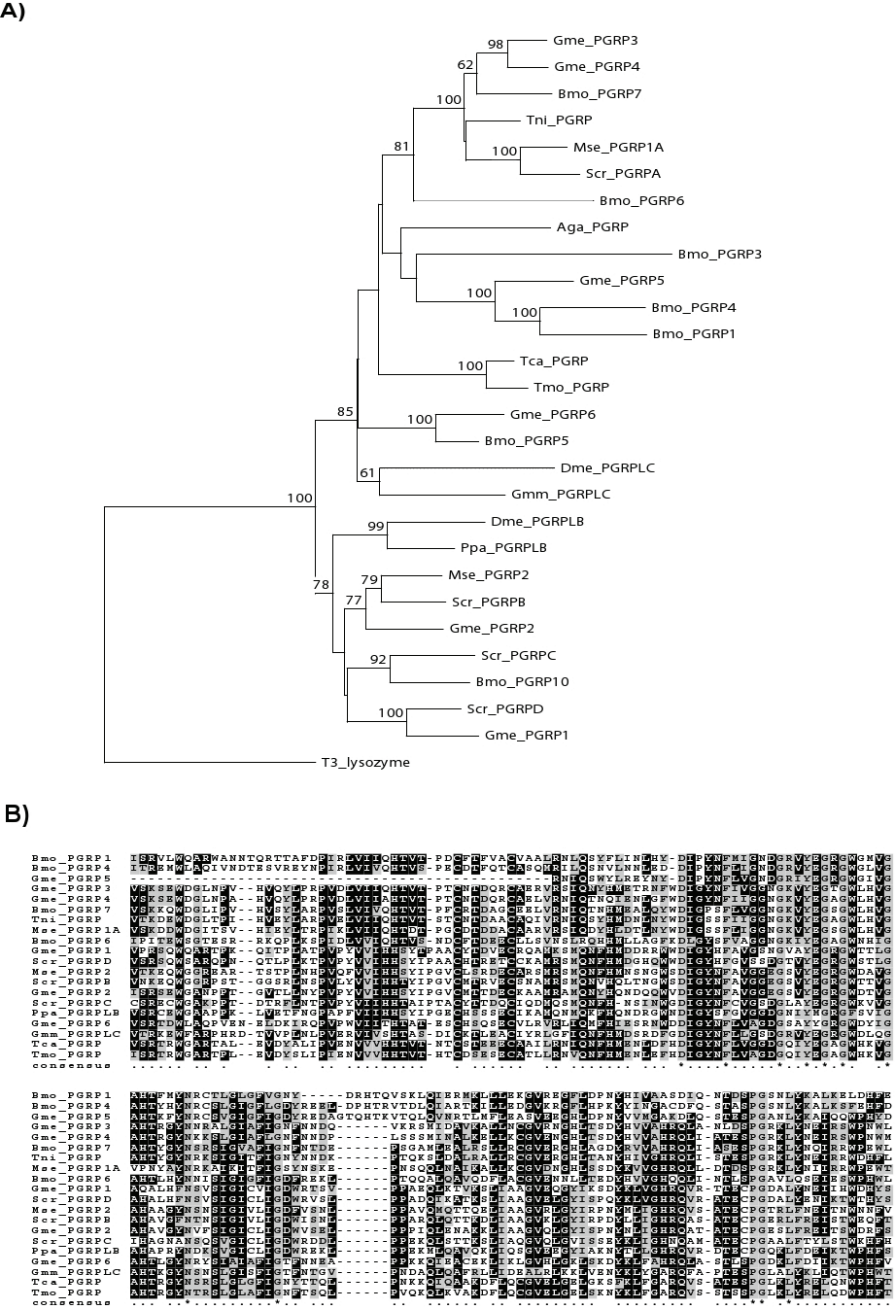
**Gene phylogeny and amino acid alignment of PGRP protein sequences**. (A) A bayesian phylogenetic tree of insect PGRP proteins. Bayesian posterior probabilities are shown for all major nodes supported with probability higher than 60%. (B) Amino acid alignment of the 6 predicted proteins from Galleria together with predicted protein sequences deduced from publicly available insect sequence datasets. Amino acid sequence alignments were performed using MAFFT multiple alignment program without the predicted signal peptide and part of the N-terminus as in some cases only partial sequence information was available. Identical residues are boxed with dark shading, and conserved residues are boxed with light shading. Species abbreviations: *Manduca sexta *(Mse), *Bombyx mori *(Bmo), *Trichoplusia ni *(Tni), *Phlebotomus papatasi *(Ppa), *Samia cynthia ricini *(Scr), *Tribolium castaneum *(Tca)*, Glossina morsitans *(Gmm), *Galleria mellonella *(Gme), *Tenebrio molitor *(Tmo).

Peptidoglycan recognition proteins (PGRPs) and apolipophorin III which are known to mediate recognition of pathogen-associated molecular patterns (PAMPs) and damage-associated molecular patterns (DAMPs) have been identified both among the determined transcripts and the new or enhanced spots of the 2D-gels, implicating their release within the hemolymph during humoral immune responses (Figure 3). Insect PGRPs specifically bind to and hydrolyze bacterial peptidoglycan, activate the Toll or IMD signal transduction pathways or proteolytic cascades that generate antimicrobial effectors, and stimulate phagocytosis. They have been found to code for up to 19 PGRPs, classified into short (S) and long (L) forms. We identified six putative PGRP sequences in *Galleria*, among which one pair solely originates from recent gene duplication (Gme_PGRP3 and Gme_PGRP4) event (Figure 4A). The two major classes of insect PGRPs are well separated in the gene phylogeny as depicted by the good bootstrap support of the Neighbour-Joining analysis. In the class which includes the gut-expressed *Drosophila *PGRP-LB gene, we can find two *Galleria *PGRPs (Figure 4B). Apolipophorin III mediates pattern recognition of beta-1,3 glucans and cellular encapsulation in *Galleria *[24]. Recently, it was shown that apoliphorin III present in the hemolymph of *Galleria *binds to nucleic acids released by damaged cells and wounded tissues, and these aggregates enhance both humoral and cellular defense reactions that can protect from infection [25].

Gram negative bacteria binding proteins (GNBPs) and β-1,3-glucan recognition proteins (βGRPs) have been extensively studied as pattern recognition proteins in Lepidoptera [26-28]. Most of these proteins are produced in the fat body and secreted into the caterpillar's hemolymph. Some are constitutively present whereas others are induced upon microbial infection. We have identified five different ßGRPs in the *Galleria *EST data collection, including one most similar to the midgut-specific short ßGRP with glucanase activity as previously described [26]. To further examine the relationships among βGRP proteins across insects and the ßGRPs identified in *Galleria*, a total of 45 sequences from 24 species, including many proteins that had previously been found in insect hemolymph, were collected and used to construct a Bayesian phylogeny (Additional File 3). The phylogenetic analysis revealed that these sequences clustered in two distinct clades. One of these clades is clearly separated from the other clades by a high posterior probability and contains the *Helicoverpa armigera *Glucanase-1 protein (described in Pauchet et al. [26]), and sequences from cDNA libraries made from midgut tissue of different Lepidoptera species, including one *Galleria *sequence. This phylogeny suggests that *Galleria *does have all of the ßGRPs found in more derived Lepidopteran species, including the gene coding for a protein with glucanase activity. This supports the idea of an ancient ßGRP duplication event in Lepidoptera, leading to paralogues that have different functions.

### Immunity related signaling

In insects, cell signaling against fungal and bacterial pathogens occurs through the Toll, Imd, and Jak-STAT pathways [29]. These pathways are quite similar to the vertebrate (e.g. TNF) signaling pathways, and induce the expression of antimicrobial peptides and other molecules through interaction with NFkB factors. The major signaling pathways Toll and Imd are represented by central receptors such as toll, toll-like, 18 wheeler and related LRR repeat-containing G-protein coupled receptors. We have identified at least three different toll or toll-like receptor transcripts in the *Galleria *dataset. The exact number of different toll receptors is not easy to evaluate, as some of the transcripts are incomplete and the predicted amino acid sequences do not always overlap. In addition to toll, we identified two different 18 wheeler partial transcripts with homology to *Spodoptera frugiperda *18 wheeler (Genbank entry ADV41489: Li, S: A 18 wheeler toll receptor gene from *S. frugiperda *cell is in response to LPS and *Saccharomyces cerevisiae *stimulation). However, a critical evaluation of the role of 18 wheeler in *Drosophila *has put its postulated function as a pattern recognition receptor for Gram negative bacteria into question [30]. Interestingly, we identified transcripts encoding for the transcription factors NFkB and relish which function as obligate dimmers. Relish regulates downstream of the IMD pathway expression of antimicrobial peptides in *Drosophila *[31]. While the signaling pathways that stimulate immune gene expression have been well characterized by genetic analysis in *Drosophila*, they are far from being well understood in most other insect species. However, several proteins involved in these pathways have recently been characterized in Lepidoptera. One such pathway involves proteolytic activation of a cytokine called Spaetzle, which functions in dorsal-ventral patterning during early embryonic development and in the antimicrobial immune response in larvae and adults. Most interestingly, it could be shown that injection of Spaetzle into *M. sexta *larvae stimulated expression of several immune-related peptides and proteins, including cecropin, attacin, moricin and lysozyme [32]. We have identified a Spaetzle homolog in *Galleria*. The Gme-Spaetzle cDNA encodes a polypeptide with 29%, 42% and 44% identity to *N. vitripennis, B. mori *and *M. sexta*, respectively (Additional file 4A-alignment of *Galleria, Bombyx, Manduca *Spaetzle).

In addition to major immune signaling proteins, we identified a calreticulin sequence in the immune-induced *Galleria *transcriptome data. Calreticulin is involved in signal transduction events associated with innate immunity, cell adhesion, angiogenesis and apoptosis in mammals. The level of calreticulin on the surface of human dentritic cells and polymorphonuclear phagocytes correlates with their phagocytotic ability [33]. Induction of calreticulin upon LPS challenge has recently been determined in other invertebrates such as the planarian *Schmidtea mediterranea *which is suggestive for its evolutionarily conserved roles in innate immunity [34]. LPS-challenge also induced expression of tetraspanins whose role in modulating immune signal complexes in vertebrates is well established [35]. Its induced expression upon LPS-injection has also been documented in ancient insects such as the firebrat [19]. Similarly, an ankyrin repeat domain containing protein was found both in this basal insect and in *Galleria*.

### Antimicrobial peptides and proteins

**Figure 5:**
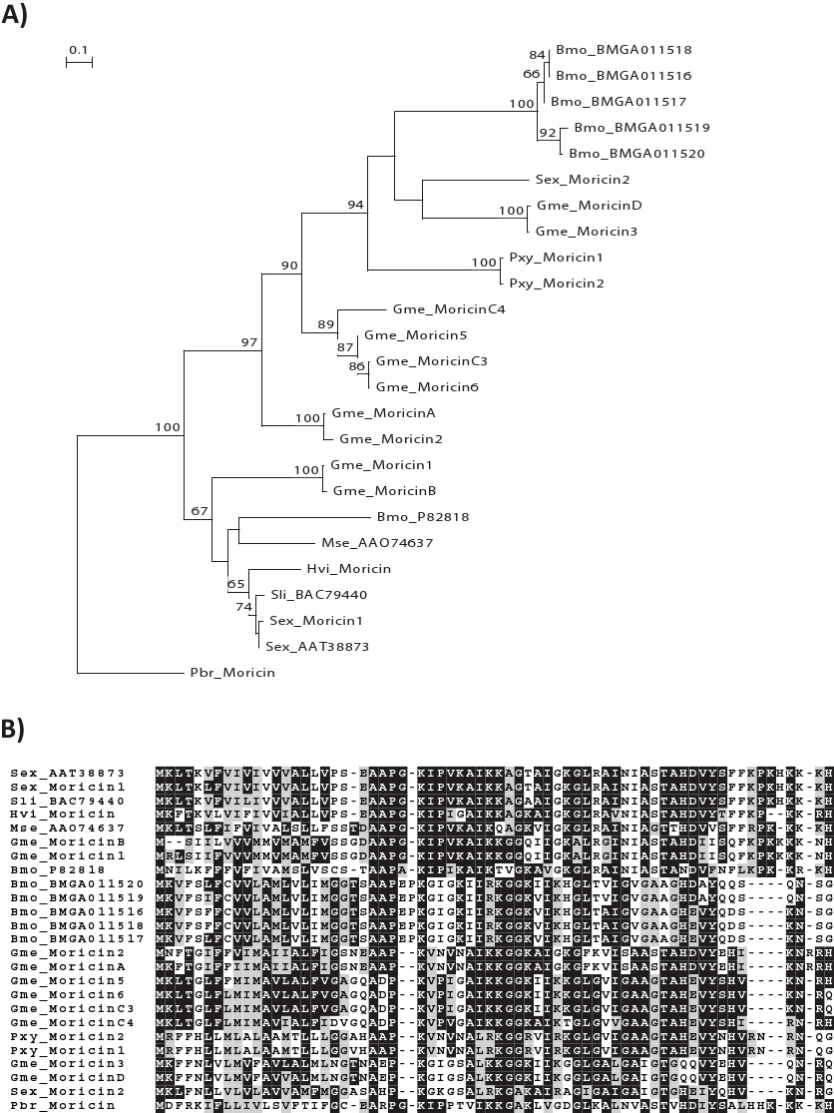
**Gene phylogeny and amino acid alignment of moricins**. (A) Neighbor-joining (NJ) consensus tree of moricins from *Galleria mellonella *(Gme) and other insect species. (A) Bootstrap values next to the nodes represent the percentage of 1000 replicate trees supporting the corresponding clade. (B) Amino acid alignment of the predicted moricin peptides from Galleria together with predicted protein sequences deduced from publicly available insect sequence datasets. Species abbreviations: *Spodoptera exigua *(Sex), *Spodoptera littoralis *(Sli), *Heliothis virescens *(Hvi), *Manduca sexta *(Mse), *Bombyx mori *(Bmo), *Plutella xylostella *(Pxy), *Pieris brassicae *(Pbr). Sequences were aligned with MAFFT multiple alignment program. Identical residues are boxed with dark shading, and conserved residues are boxed with light shading.

**Figure 6:**
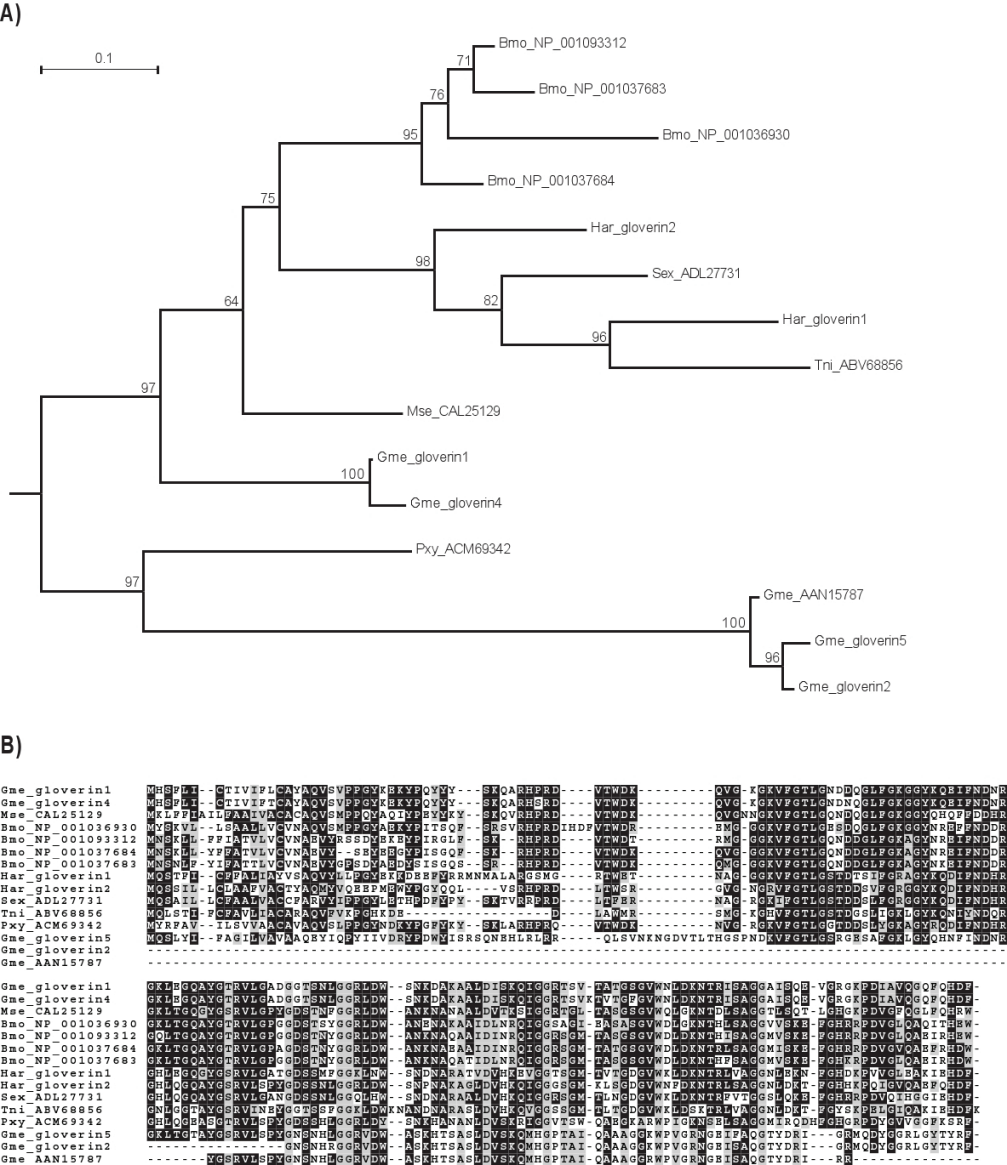
**Gloverin gene phylogeny and amino acid alignment**. (A) Bootstrap values next to the nodes represent the percentage of 1000 replicate trees supporting the corresponding clade. (B) The predicted gloverin proteins from Galleria (Gme) were aligned together with other lepidopteran gloverin proteinsusing MAFFT multiple alignment program. Identical residues are boxed with dark shading, and conserved residues are boxed with light shading. Sequences Gme_gloverin2 and 5 are almost identical to the (partial) gloverin sequence deposited in Genbank (AAN15787). Abbreviated species names: *Manduca sexta *(Mse), *Bombyx mori *(Bmo), *Spodoptera exigua *(Sex), *Helicoverpa armigera *(Har), *Trichoplusia ni *(Tni), *Plutella xylostella *(Pxy).

Our transcriptomic analysis resulted in identification of a large number of antimicrobial peptides and proteins (AMPs) among which the moricin-like gene family, the gloverins and the cecropins were prominent. We determined the presence of six genes coding for moricin-like proteins of the eight moricin peptide fragments (several of which are identical) reported from *Galleria*. Moricins have been shown to exert *in vitro *activity against both Gram negative and Gram positive bacteria, as well as against yeast and filamentous fungi [36]. Although protein sequence alignments show highly conserved blocks of amino acids, a phylogenetic analysis of moricin sequences from *Galleria *and other Lepidoptera indicate species-specific gene duplication events for some gene family members (e.g. most of the *Bombyx *moricins), while others cluster according to the species phylogeny (Figure 5). Moricins belong to the amphipathic α-helical antimicrobial peptides and have been first discovered in the lepidopteran *B. mori *[37], while gloverins have first been found in the silk moth *Hyalophora gloveri *[38]. The presence of moricins and gloverins seem to be restricted to Lepidoptera. Both proteomic and transcriptomic analysis confirmed the induced expression of gloverins and their secretion into the hemolymoh of in *Galleria *(Figure 3). We identified five members among the induced transcripts. Gloverins are basic and heat-stable proteins enriched in glycine residues but lacking cysteine residues (Figure 6). They interact with LPS and thereby increase the permeability and inhibit the formation of the outer membrane in bacteria. At least 7 gloverins have been reported from the genome of the silkworm *B. mori *[39].

Cecropins represent another group of linear and amphipathic peptides with a-helical structure. The first member of this peptide family exhibiting antibacterial and antifungal activity was discovered in and isolated from the hemolymph of the silk moth *Hyalophora cecropia *and has therefore been named cecropin [40]. The cecropin-like peptide from *Galleria *is synthesized as a propeptide, with a putative 22-residue signal peptide, a 4-residue propeptide and a 39-residue mature peptide with a mass of 4.3 kDa. Like cecropins from other insects it exhibits potent activity against both Gram-positive and Gram-negative bacteria [41]. We have identified four different cecropins in the *Galleria *transcriptome dataset, including a more diverged D-type cecropin. This surprisingly large number of different cecropins (Additional file 4B) covers a larger fraction of the amino acid diversity encountered when comparing cecropins from across the Lepidoptera.

We determined both cysteine-rich peptides reported from *Galleria *which exclusively inhibit growth of filamentous fungi, the defensin-like antifungal peptides galiomicin [42] and gallerimycin [43]. At least the latter contributes to innate immune responses mediating resistance of *G. mellonella *larvae against normally lethal infection by the human pathogenic yeast *C. albicans *[14]. Transgenic expression of gallerimycin has been shown to confer resistance to fungal diseases on crops [43]. A homologue of spodoptericin, the third defensin-like peptide discovered in Lepidoptera [44], is also present in our *Galleria *transcriptome.

In a previous study, we used the suppression subtractive hybridization method to screen for genes that are induced in *Galleria *upon challenge with LPS [18]. This approach resulted in the discovery of novel peptides and protein families which were also found in this extended transcriptomic study. For example, we discovered a cobatoxin-like molecule and a protein which was named Gall-6-tox due to its six conserved tandem repeats of cysteine-stabilized alpha beta motifs (CS-αβ), the structural scaffold characteristic of invertebrate defensins and scorpion toxins. Homologues of Gal-6-tox differing in the number of tandem repeats of the CS-αβ motif were later found in other lepidopterans such as *Bombyx mori *and *Spodoptera exigua*. It turned out that they belong to a novel family of atypical defensin-derived immune-related proteins, which is specific to Lepidoptera and which is now called X-tox [45]. Moreover, our study confirmed the induced expression of tenascin-like proteins in *Galleria *upon LPS-challenge [18], which represent immune effector molecules known from vertebrates. However, using RACE-PCR we obtained the full-length cDNA which is considerably shorter than vertebrate tenascins and lacks characteristic tenascin domains such as fibronectin type-3-like repeats. These findings make the relation of the identified sequences to tenascins unlikely.

**Figure 7:**
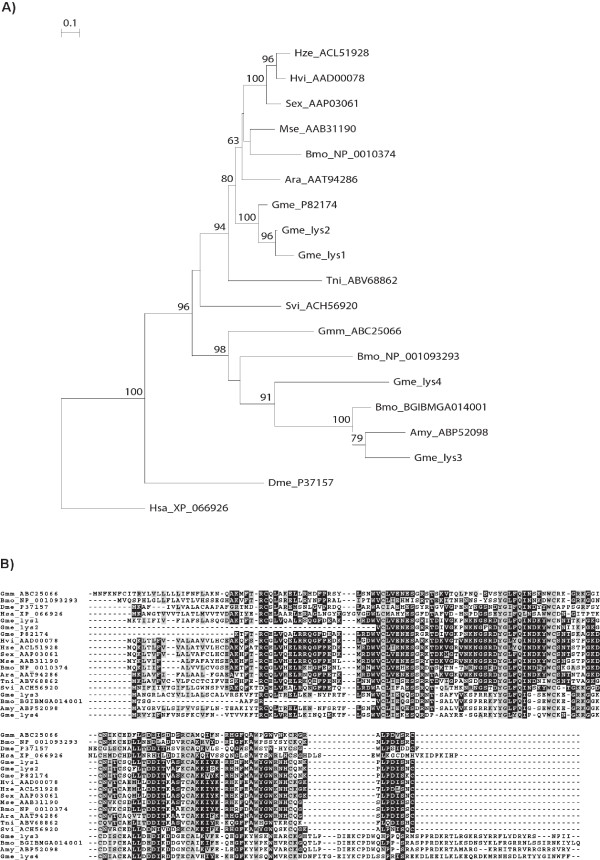
**Gene phylogeny and sequence similarity of C-type Lysozymes**. Neighbour-joining phylogenetic analysis of C-type lysozymes (C-Lys) from *Galleria mellonella *(Gme), other insect species and humans (accession numbers are provided in the name IDs). (A) Bootstrap values next to the nodes represent the percentage of 1000 replicate trees supporting the corresponding clade. (B) Amino acid alignment of the five predicted C-type lysozyme proteins from Galleria together with predicted protein sequences deduced from publicly available insect sequence datasets.

With transcriptomic and proteomic analysis we also found different lysozymes, one of which was first identified in *Galleria *more than 40 years ago, representing the first antimicrobial protein reported from insects [46]. It shares structural similarity with C (chicken) type lysozyme [47], and its activity against Gram-positive bacteria has been attributed to its ability to degrade cell wall peptidoglycan by hydrolysis of the b-1-4 linkages between N-acetylglucosamine and N-acetylmuramic acid residues. Besides moderate activity against Gram-negative bacteria [48], *Galleria *lysozyme was also shown to exhibit antifungal activity *in vitro *[49], similar to that of human lysozyme against the pathogenic yeasts *Candida albicans *and *Coccidioides immitis *[50]. We identified four c-type lysozyme homologues and an additional i-type lysozyme whose function remains to be elucidated. To further examine the relationships among lysozyme proteins identified in *Galleria *and those found in other insects, c-type lysozyme sequences from 12 insect species and from human were aligned and used to construct a gene phylogeny (Figure 7A). The phylogenetic analysis revealed that these sequences clustered in two distinct clades. One of these clades clearly separated with a high bootstrap support contains most of the lepidopteran lysozymes, including a group with two of the four *Galleria *lysozymes identified here and a previously identified partial lysozyme sequence (Figure 7B). These findings suggest that *Galleria *exceeds the number of c-type lysozymes found in other Lepidoptera (e.g. three lysozymes identified in the genome of *Bombyx*), supporting the idea of species-specific lysozyme gene duplication events in *Galleria *leading to paralogues with potentially different functions.

**Figure 8:**
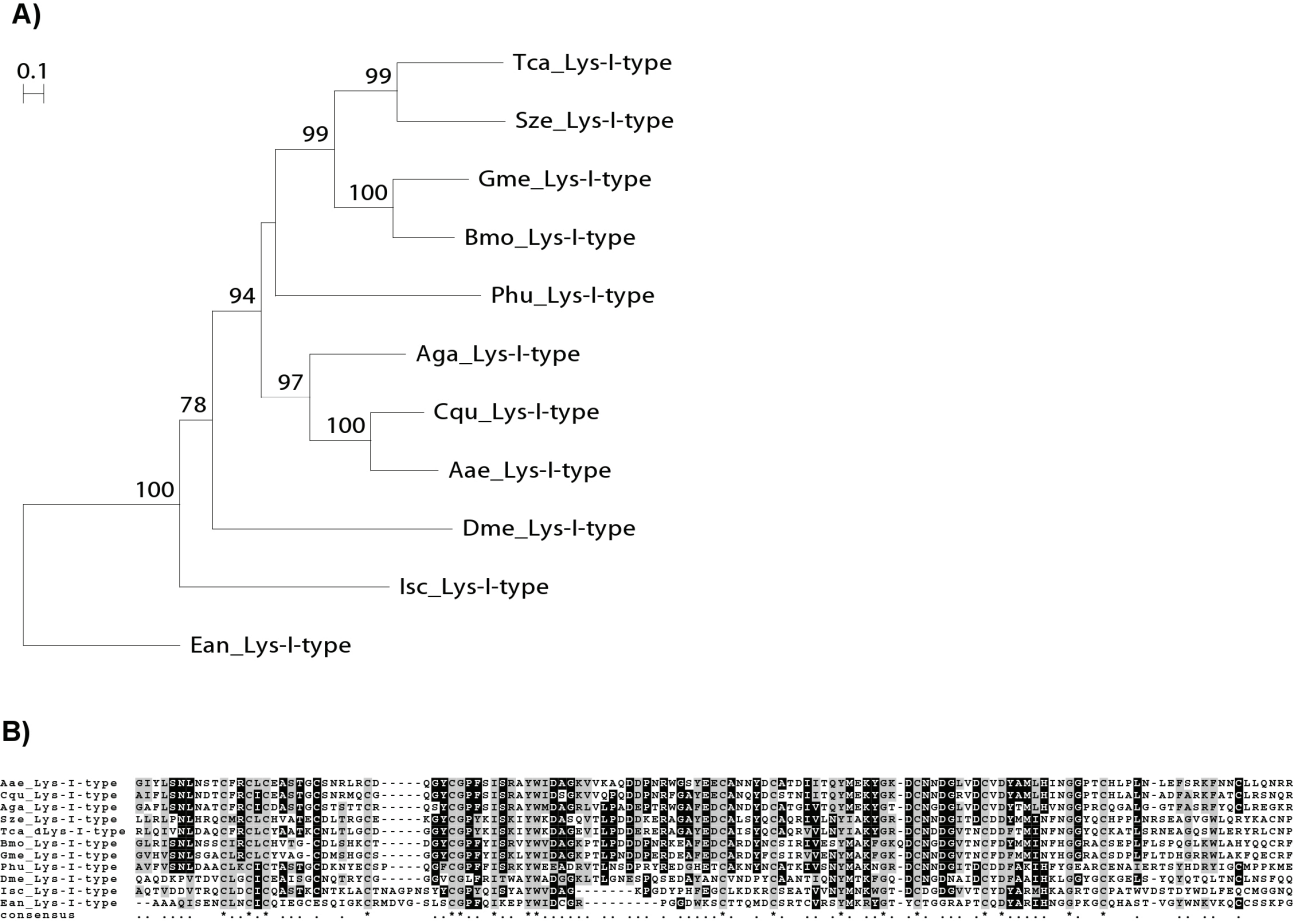
**Gene phylogeny and amino acid alignment of i-type Lysozymes**. (A) Neighbour-joining (NJ) phylogenetic analysis of i-type lysozymes from *Galleria mellonella *(Gme), other insect and non-vertebrate species (accession numbers are provided in the name IDs). Bootstrap values from NJ analyses are shown as percentages of 1000 replicate trees supporting the corresponding clade. (B) MAFFT alignment with part of the predicted i-type lysozyme proteins listed in the phylogeny. Identical amino acids are shaded in black and depicted by an asterisc, conserved amino acids are shaded in grey and depicted by a dot in the consensus sequence.

The invertebrate i-type lysozymes, although somewhat diverged in their activities, encompass a group of proteins having highly related primary structures. They differ from other lysozymes in having 10 or 12 cysteine residues in the primary sequence. The latter are predicted to form five or six disulfide bonds which have been attributed to cause stability against heat denaturation, high osmolarity and proteolytic degradation. Although several i-type lysozymes have been shown to be active at low temperatures, the enzyme is stabile even after prolonged heating or long-term storage at room temperature [51]. The i-type lysozymes are coded for by single copy genes in Lepidoptera (Figure 8).

In addition, we identified a full-length cDNA sequence which is identical to the deduced protein sequence of a *Galleria *proline-rich peptide [52] and almost identical to two protein fragments identified in a previous study analyzing hemolymph peptide fragments in *Galleria *[53]. Finally, our transcriptomic analysis confirmed the presence of genes encoding cobatoxin-like peptides [18,45] (Additional file 4C).

### Inhibitors of microbial proteases

**Figure 9:**
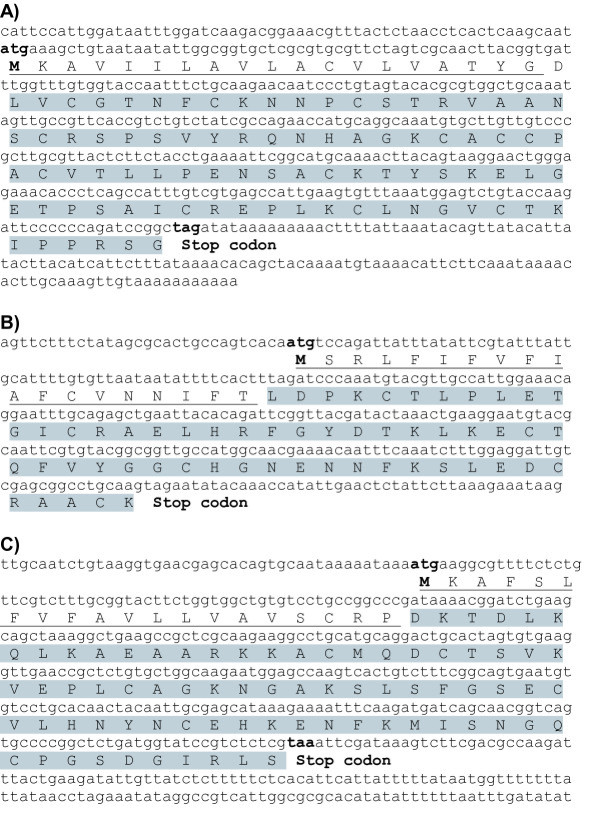
**Nucleotide and amino acid sequences of three insect serine protease inhibitors (ISPIs) in *Galleria***. Three identified cDNAs are shown including open reading frames that encode *Galleria *ISPI1 (A), ISPI2 (B) or ISPI3 (C). Start methionine and stop codons are indicated. Signal sequences predicted with ProP 1.0 Server http://www.cbs.dtu.dk/services/ProP/ are underlined and active peptide sequences are shaded.

The immunity-related transcriptome of *Galleria *comprises a number of serine proteinase inhibitors among which we identified three genes encoding inhibitors (ISPI-1, ISPI-2, and ISPI-3) that have previously been purified from hemolymph and whose amino acid sequence was partially determined by Edman sequencing. All three ISPIs with molecular masses between 9.2 (ISPI-1) and 6.3 (ISPI-3) were determined to be capable of inhibiting the major virulence factors, designated as Pr1 and Pr2, of the entomopathogenic fungus *Metarhizium anisopliae *which is used in biological control of pest insects worldwide [54]. ISPI-1 and ISPI-3 do not share sequence similarity with other known proteins whereas ISPI-2 turned out to represent a Kunitz-type inhibitor (Figure 9). In addition, we found transcripts encoding for the insect metalloproteinase inhibitor (IMPI) which represents the first and to date only peptide known from animals which is capable to specifically inhibit thermolysin-like microbial metalloproteinases belonging to the M4 family [55]. The latter encompass a number of prominent members such as aureolysin, bacillolysin, pseudolysin and vibriolysin which are produced by human pathogenic bacteria. Thermolysin-like metalloproteases are recognized to be responsible for a number of symptoms associated with severe infections such as increase of vascular permeability, hemorrhagic edema, sepsis and necrotic tissue destruction in infected humans, and have therefore been implicated as targets for the development of second generation antibiotics [56]. The IMPI has been discovered in and was purified from *Galleria *larvae which were preinjected with LPS [57]. The amino acid sequence of the IMPI shares no similarity with other known proteins and its prominent stability against heat and acid treatment has been attributed to its five intra-molecular disulfide bonds. It has recently been found to encode two distinct inhibitors. The IMPI-peptide encoded by the N-terminal part contributes to innate immune responses by inhibiting microbial metalloproteases, whereas the IMPI-peptide encoded by the C-terminal part of the gene has been implicated to mediate regulation of endogenous matrix metalloproteinases with pleiotropic functions in immunity and development [58].

### Transferrin

Several induced transcripts encode for transferrin which represents a multifunctional and evolutionarily conserved player in innate immunity. Its role in binding and removing available free iron ions, thus creating unfavorable environments for bacteria has first been reported in vertebrates [59]. A recent study using *B. mori *confirmed both the induced expression of transferrin upon LPS-challenge and its contribution to antibacterial iron-withholding strategy in Lepidoptera *B. mori *[60].

### Stress response genes

In line with our previous studies in which we used LPS-challenge to screen for inducible immunity-related genes in insects and other invertebrates [18-20,29,61] we determined induced expression of genes involved in detoxification and stress adaptation such as apolipoprotein D, cytochrome P450s, gluthathione S-transferases, and a number of heat shock proteins which further supports our hypothesis that interdependencies between immune and stress responses are evolutionarily conserved in insects [18-20,29,61]. Glutathione S-transferases (GSTs) are a large and diverse family of detoxification enzymes found in most organisms. GSTs help to protect cells from oxidative stress, but they also play a central role in the detoxification of both endogenous and xenobiotic compounds (e.g. plant secondary metabolites or insecticides) and are involved in intracellular transport and biosynthesis of hormones. Eukaryotes contain multiple GSTs belonging to different GST classes and with differing enzyme activities to accommodate the wide range of functions of this enzyme family. The insect GST supergene family encodes a group of proteins that have been assigned to at least six classes: Delta, Epsilon, Omega, Sigma, Theta and Zeta [62,63]. The Delta and Epsilon classes, both specific to insects, are the largest classes and are often involved in xenobiotic metabolism whereas the Omega, Sigma, Theta and Zeta classes have a much wider taxonomic distribution and likely play essential housekeeping roles [62,63]. Herbivorous insects have to cope with toxic plant metabolites taken up with their diet and GSTs can play an important role in their detoxification [64-66].

We identified a total of 19 different GSTs in *Galleria *larval ESTs out of which 2 were microsomal GSTs. Five out of the six classes identified in other insect species are represented and most of the Galleria GSTs belong to the insect-specific Delta and Epsilon classes with 4 and 6 members each, respectively. However, in contrast to a comparable larval EST dataset of the generalist plant herbivore lepidopteran *H. armigera *[67] both the total number of GSTs identified and the strong overrepresentation of the insect-specific GSTs is much lower in *Galleria *(Additional file 5). The insect-specific Delta and Epsilon GST classes are often involved in detoxification of xenobiotics and the limited number of GSTs from those classes may point at the unique ecological niche and highly specialized diet of Galleria which is devoid of any (potentially toxic) plant secondary metabolites.

## Conclusions

We have generated a comprehensive larval transcriptome map of the phylogenetically ancient lepidopteran *Galleria mellonella*. This data set complements and massively expands the known spectrum of immunity and stress related genes of this model host which have been found in previous studies using peptidomic [68] or SSH-based transcriptomic approaches [18]. Besides genes encoding proteins that mediate recognition of microbial signatures such as GNBPs, βGRPs, PGRPs and Toll or immunity-related signalling, we determined a broad spectrum of defence related effectors such as antimicrobial peptides and proteins among which moricins and gloverins are restricted to Lepidoptera. In line with other studies, the spectrum of genes which is up-regulated in response to injected LPS includes proteins involved in detoxification (apolipoprotein D, cytochrome P450s, gluthathione S-transferases) and stress response (e.g. heat shock proteins). The secretion of induced immunity-and stress-related peptides and proteins into the hemolymph has been confirmed by comparative proteomic analysis of hemolymph samples from untreated and immunized larvae. Importantly, the spectrum of immunity-related genes identified in this study shares high similarity with that found in another lepidopteran species, the tobacco hornworm *M. sexta*, whose killed bacteria-induced transcriptome has previously been analyzed by pyrosequencing [1]. Furthermore, except for attacins, we identified in *Galleria *members of all families of antimicrobial peptides which are predicted from the complete genome sequence of *B. mori*, [39]. Taken together we postulate that all effector molecule families contributing to lepidopteran innate immunity are present in the phylogenetically basal family Pyralidae to which *Galleria *belongs. The entity of generated data provide a valuable platform for more detailed analyses of immune responses in *Galleria *and, therefore, improve the suitability of this lepidopteran both as a model host for human pathogens and for studies addressing coevolution with entomopathogens.

## Methods

### Insect material

*Galleria mellonella *individuals used here were obtained from the laboratory culture which has been used in our previous studies. *Galleria *caterpillars were reared on an artificial diet (22% maize meal, 22% wheat germ, 11% dry yeast, 17.5% bee wax, 11% honey, and 11% glycerin) at 31°C in darkness. Last-instar larvae, each weighing between 250 and 350 mg, were used for immunization using 10 mg/ml LPS dissolved in water (Sigma, Taufkirchen, Germany). Ten microliters of sample volume per caterpillar was injected dorsolaterally into the hemocoel using 1-ml disposable syringes and 0.4-by 20-mm needles mounted on a microapplicator. Larvae were homogenized at 8 h postinjection for RNA isolation or bled at 24 h postinjection to obtain hemolymph samples.

### RNA extraction, cDNA normalization and Next Generation Sequencing

Total RNA was extracted from different life stages, from hemocytes, and from immune-challenged larvae (injections) using TRIZOL and mRNA was subsequently isolated from total RNA using the MN-NucleoTrap mRNA kit according to the manufacturers' instructions (Macherey & Nagel). cDNAs were generated from 1 μg of poly(A)+ mRNA using the SMART PCR cDNA synthesis kit (BD Clontech) following the manufacturer's protocol. Reverse transcription was performed with the SMART KIT reverse transcriptase (Takara) for 60 min at 42°C. In order to prevent over-representation of the most common transcripts, the resulting single-stranded cDNAs were normalized using the Kamchatka crab duplex-specific nuclease method (Trimmer cDNA normalization kit, Evrogen) [69]. Subsequently, SMART kit components and Triple-Taq enzyme with proof-reading activity were used to generate full-length enriched double-stranded long cDNAs. Each step of the normalization procedure was carefully monitored to avoid the generation of artefacts and overcycling. The optimal condition for ds-cDNA synthesis was empirically determined by subjecting the cDNA to a range of thermocycling numbers and their products checked by electrophoresis. The optimal cycle number was defined as the maximum number of PCR cycles without any signs of overcycling. The resulting normalized cDNA library was used for 454 pyrosequencing [70] using the Roche 454 FLX machine and Sanger sequencing using an ABI 3730 × l capillary sequencer. The 454 sequence reads were assembled using the newbler assembler with standard settings and using the CLC Genomics Workbench as an alternative assembly method. Before assembly, obtained reads were preprocessed by masking PolyA tails and removing SMART adapters using custom written Perl scripts. We compared the resulting contigs to the refseq protein database containing all information on coding sequences so far obtained (March 2010). Furthermore, we set up species specific databases from *Drosophila, Bombyx*, and human in order to find species specific similarities.

### Sanger Sequencing and Generation of EST Databases

A fraction of the dscDNAs was cloned in the pGEM-T-easy vector. Ligations were transformed into *E. coli *ELECTROMAX DH5α-E electro-competent cells (Invitrogen). Plasmid minipreparation from bacterial colonies grown in 96 deep-well plates was performed using the 96 well robot plasmid isolation kit (NextTec) on a Tecan Evo Freedom 150 robotic platform (Tecan). Sequencing of both the 5' and 3' termini of cDNA library clones was carried out on an ABI 3730 xl automatic DNA sequencer (PE Applied Biosystems). Vector clipping, quality trimming and sequence assembly using stringent conditions (e.g. high quality sequence trimming parameters, 95% sequence identity cutoff, 25 bp overlap) was done with the Lasergene software package (DNAStar Inc.).

### Blast homology searches and sequence annotation

We set up individual searchable databases for the complete sequence dataset and used this to identify the genes we describe in more detail in the text. Blast searches were conducted on a local server using the National Center for Biotechnology Information (NCBI) blastall program. Homology searches (BLASTx and BLASTn) of unique sequences and functional annotation by gene ontology terms (GO; http://www.geneontology.org), InterPro terms (InterProScan, EBI), enzyme classification codes (EC), and metabolic pathways (KEGG, Kyoto Encyclopedia of Genes and Genomes) were determined using the BLAST2GO software suite v2.3.1 http://www.blast2go.de[71]. Homology searches were performed remotely on the NCBI server through QBLAST, and followed a sequential strategy. First, Sequences were searched against the NCBI non-redundant (nr) protein database using an E-value cut-off of 10-3, with predicted polypeptides of a minimum length of 15 amino acids. Second, sequences retrieving no BLASTx hit were searched again by BLASTn, against an NCBI nr nucleotide database using an E-value cut-off of 10-10. The GO data presented represent the level 3 analysis, illustrating general functional categories. Enzyme classification codes, and KEGG metabolic pathway annotations, were generated from the direct mapping of GO terms to their enzyme code equivalents. Finally, InterPro searches were performed remotely from BLAST2GO via the InterProEBI web server. In order to obtain a rough transcriptome coverage estimate for the *Galleria *larval cDNA library, we went through a series of search steps in order to i) obtain all hits against the conserved KEGG pathway database, and ii) estimate genome coverage by identifying the complete ribosomal protein dataset as compared to the full *B. mori *set. Based on these findings we estimate the theoretical transcriptome coverage to be close to 90% (e.g. 77/79 *B. mori *ribosomal proteins were found). Nucleotide sequences were analyzed in more detail using the commercial Lasergene Software package and the freeware BioEdit program. Genes were aligned by their amino acid sequences using the ClustalW function [72] or the MAFFT program. If necessary, alignments were then corrected by eye and reverted back to the nucleotide sequences for the phylogenetic analyses and in order to remove redundant contigs.

### Sequence submission

We have deposited the EST (Sanger) and short read (454 Roche) data with the following accession numbers: ERP000555 (SRA) and JG394435-JG406465 (dbEST). Phylogenetic data was deposited at TreeBASE with submission ID 11389. All of the predicted protein sequences used for alignments and phylogenies can be found in additional file 6. Note that the names of the validated proteins are made from the letters Gme followed by the number of the contig from the assembly. An assembly of the *Galleria *data with contig consensus sequences, Blast2GO hits against nr database, hit accessions, and annotations including InterPro scans can be found in Additional file 7.

### Phylogenetic reconstruction

The phylogenetic reconstruction implemented for the analysis of several proteins was performed using two different methods. For the Neighbour-Joining (NJ) method we implemented the TREECON program. Amino acid sequences were aligned by MAFFT http://mafft.cbrc.jp/alignment/server/index.html and each visually inspected for regions of high quality alignment. The NJ consensus tree was generated with TREECON. Distance calculations were performed after Tajima & Nei and bootstrap analysis, running 1000 bootstrap samples. Conserved residues in the alignments were highlighted with BOXSHADE 3.21 http://www.ch.embnet.org/software/BOX_form.html. In addition to the Neighbour-Joining method, for some gene trees the phylogenetic reconstruction was done by Bayesian inference using Mr. Bayes 3.1. The prior was set for the amino acid models to mix, thereby allowing model jumping between fixed-rate amino acid models. Markov Chain Monte Carlo runs were carried out for 10,000,000 generations after which log likelihood values showed that equilibrium had been reached after the first 5000 generations in all cases, and those data were discarded from each run and considered as 'burnin'. Two runs were conducted for the dataset showing agreement in topology and likelihood scores. The Neighbour-joining and the Bayesian tree topologies including their general subfamily relationships and node supports were in agreement. The gene trees were visualized and optimized with the MEGA4 software package [73].

### Two-dimensional gel electrophoresis of hemolymph proteins

Proteomic analysis of immune hemolymph has been performed as described previously [21]. In brief, hemolymph samples from 10 larvae 24 h post immune challenge and from 10 untreated larvae used as controls were collected directly into 1.5 ml pre-cooled plastic tubes containing traces of phenylthiourea to prevent melanisation reactions. Hemocytes were removed by brief centrifugation step and cell-free hemolymph was precipitated by the addition of 3 volumes of 100% acetone and 0.4 volumes of 100% trichloroacetic acid and incubation at 20°C for 1 h. After centrifugation at 20,000 × *g *for 10 min, the pellet was washed three times with 100% acetone and resolved under agitation in 8 M urea at 22°C for 16 h. Protein concentrations were determined using a Micro BC assay kit (Uptima, Montlucon, France). Two-dimensional gel electrophoresis was done with the Ettan IPGphor II system and the Ettan DALTsix electrophoresis unit (Amersham Biosciences, Uppsala, Sweden) according to the instructions of the manufacturer. Briefly, 1 mg of protein was mixed with immobilized pH gradient (IPG) buffer (pH 3 to 11 nonlinear gradient [NL]) and applied on an IPG strip (24 cm; pH 3 to 11 NL). Isoelectric focusing was performed at 20°C and 75 μA per IPG strip as follows: swelling for 24 h and isoelectric focusing for 1 h at 500 V, 8-h gradient to reach 1,000 V, 3-h gradient to reach 8,000 V, and isoelectric focusing for 4 h at 8,000 V. Prior to Tris-Tricine-sodium dodecyl sulfate (SDS)-polyacrylamide gel electrophoresis (5) with 26-by 20-cm 15% gels, the strips were equilibrated with 6 M urea, 30% glycerin, 2% SDS, and 50 mM Tris-HCl at pH 8 for 30 min. After electrophoresis at 20°C, the gels were stained using colloidal Coomassie brilliant blue (Carl Roth). For image analysis, the gels were scanned using an Umax PowerLook II scanner and analyzed with Delta2D software (Decodon, Greifswald, Germany). Spot identity has been determined by comparing spots with our recent study [21] combined with additional peptide mass fingerprinting analyses. In brief, spots excised from the gel were carbamidomethylated and in-gel digested using mass spectrometry grade trypsin (Promega) in 0.025 M NH_4_HCO_3_. The mass spectra of the resulting tryptic peptides were recorded using an Ultraflex TOF/TOF mass spectrometer (Bruker Daltonik, Bremen, Germany) operating under FlexControl 2.4 (Bruker) in the positive-ion reflectron mode, with dihydroxy benzoic acid as the matrix. Peptide mass profiles were analyzed with local Mascot http://www.matrixscience.com, using deduced protein sequences from our present *Galleria *transcriptome analysis database.

## Authors' contributions

AV, HV and BA conceived the study. HV analyzed data, performed sequence alignments and phylogenetic analyses, and participated in drafting the manuscript. BA performed experimental work for the generation of the cDNA library, analyzed part of the data and performed proteomic analysis of hemolymph samples. GG generated the 454 data and was involved in carrying out the bioinformatic analyses. AV conceived and coordinated the study, analyzed data and wrote the manuscript. All authors contributed in the conception and design of the study, read and approved the final version of the manuscript.

## Supplementary Material

Additional file 1 **Species distribution of the top BLAST hit in the nr database for each contig of the *Galleria *transcriptome**. Click here for file

Additional file 2 **Comparison of GO category representations between *Bombyx mori *(predicted genes) and *Galleria mellonella *transcriptome data. Each transcript was assigned applicable high-level generic GO terms. Data are presented for Biological Process and Molecular GO-level 3. Note that one gene object can be classified into more than 1 class, therefore the total number of gene objects classified for both species is not identical to the number of contigs with GO associations**. Click here for file

Additional file 3 **Gene phylogeny of ßGRP protein sequences**. A bayesian phylogenetic tree of insect ßGRP proteins. Bayesian posterior probabilities are shown for all major nodes supported with probability higher than 60%. Amino acid sequence alignments were performed using MAFFT multiple alignment program without the predicted signal peptide and part of the N-terminus as in some cases only partial sequence information was available. Identical residues are boxed with dark shading, and conserved residues are boxed with light shading. All Galleria ßGRP sequences are depicted in red and the group of ßGRP sequences with beta-glucanase activity is shaded. (AdditionalFile-3.pdf) Click here for file

Additional file 4 **Amino acid alignments of (A) Spaetzle, (B) Cecropins and (C) cobatoxin sequences from *Galleria *and other insect species**. Deduced from conceptual translation of Galleria transcripts (ESTs) present in the larval dataset. All alignments were performed with MAFFT. Identical residues are boxed with dark shading, and conserved residues are boxed with light shading. Click here for file

Additional file 5 **Gene phylogeny of glutathione S-transferases (GSTs)**. Neighbour-joining phylogenetic analysis of glutathione-S-transferases from *Galleria mellonella *(Gme) and other insect species (accession numbers are given). Bootstrap values next to the nodes represent the percentage of 1000 replicate trees that preserved the corresponding clade. Positions containing alignment gaps and missing data were eliminated and not used for the generation of the phylogenetic analysis. An additional Bayesian analysis supported all major nodes with posterior probabilities higher than 60%. Click here for file

Additional file 6 **Deduced protein sequences from *Galleria mellonella*, other Lepidopteran ESTs and NCBI sequences used in the phylogenetic analyses**. Click here for file

Additional file 7 **Complete annotation file of the assembled Galleria ESTs**. Contig IDs, sequence length, Galleria contig sequences, top BLAST hits (if any) in the NCBI nr database for each unique contig, including accession number, E-value and percentage similarity, EC numbers, GO annotations and InterPro scans are listed. Click here for file
