# Supplementary figures and images for: A comprehensive transcriptome and immune-gene repertoire of the lepidopteran model host Galleria mellonella
Source: BMC Genomics. 2011 Jun 11;12:308. doi: 10.1186/1471-2164-12-308 (PMC3224240; doi:10.1186/1471-2164-12-308)

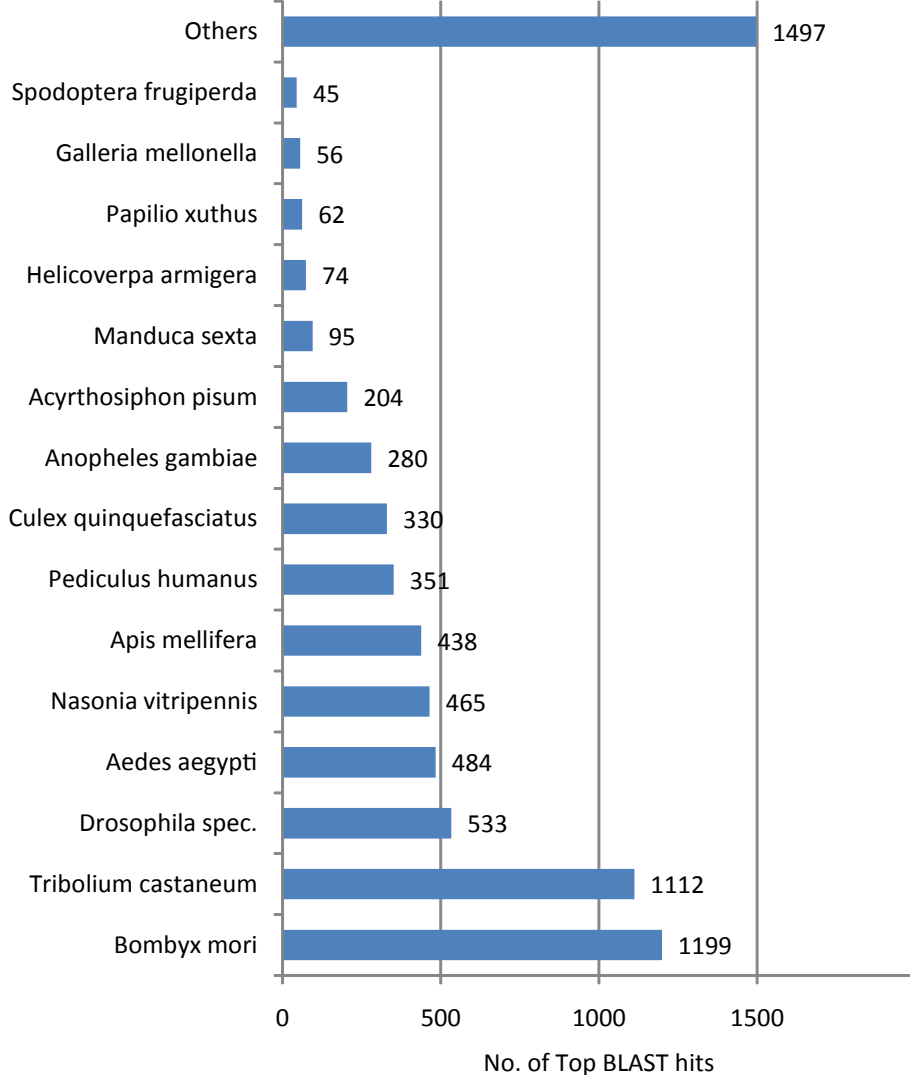

Supplement: Additional file 1 — Species distribution of the top BLAST hit in the nr database for each contig of the Galleria transcriptome. [file 1471-2164-12-308-S1.PDF]

A)

## Biological Process

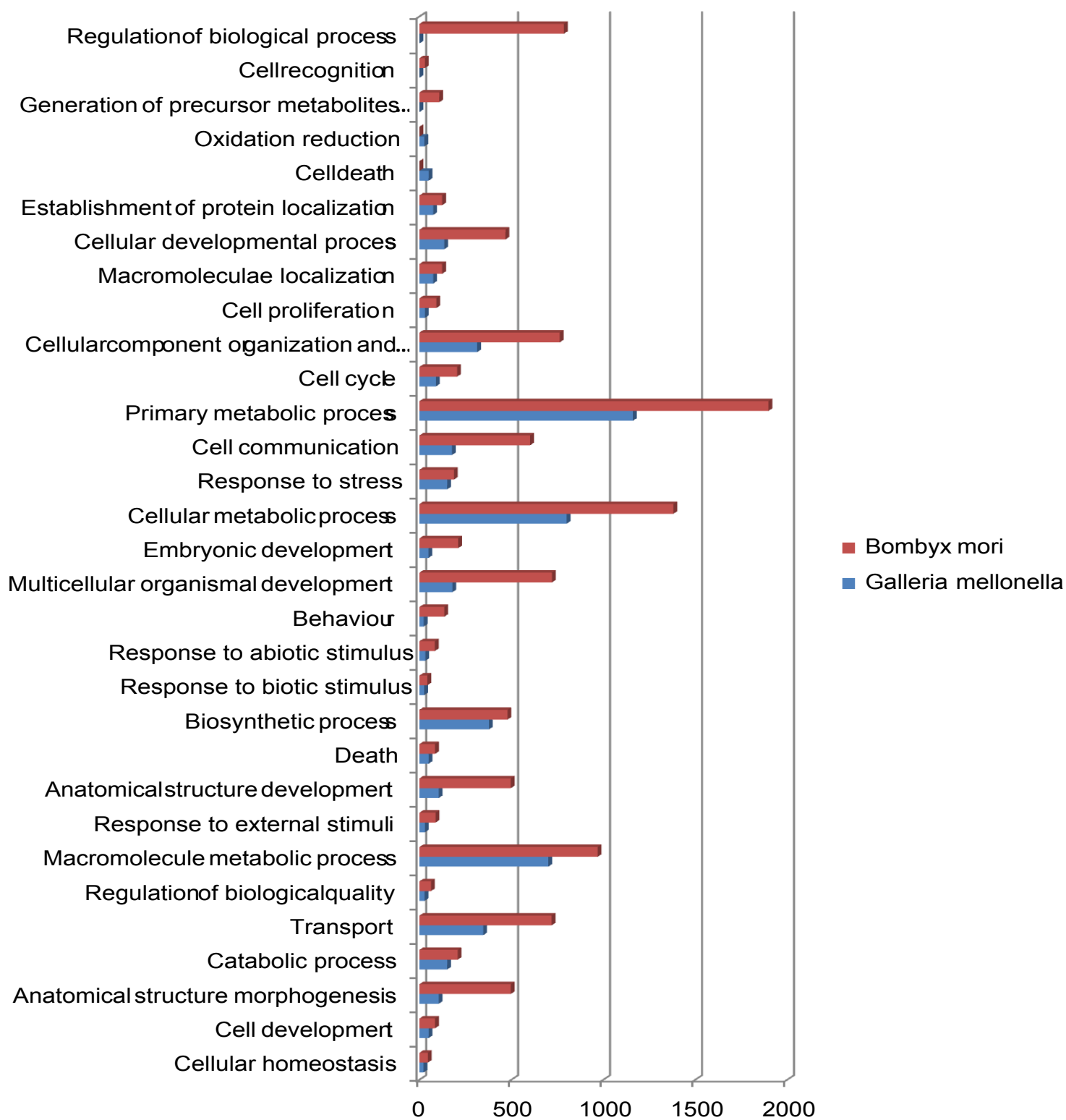

B)

## Molecular Function

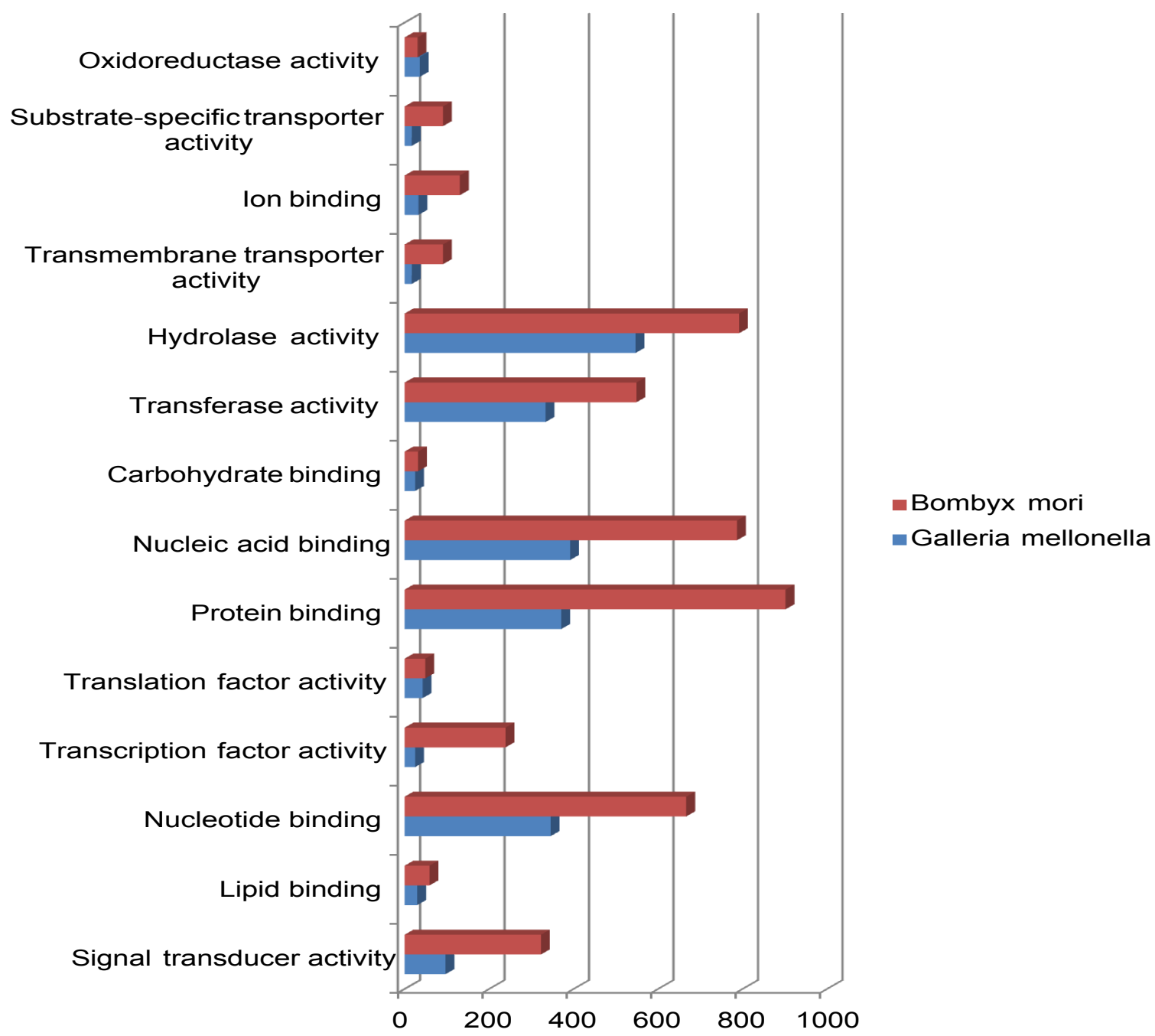

Supplement: Additional file 2 — Comparison of GO category representations between Bombyx mori (predicted genes) and Galleria mellonella transcriptome data. Each transcript was assigned applicable high-level generic GO terms. Data are presented for Biological Process and Molecular GO-level 3. Note that one gene object can be classified into more than 1 class, therefore the total number of gene objects classified for both species is not identical to the number of contigs with GO associations. [file 1471-2164-12-308-S2.PDF]

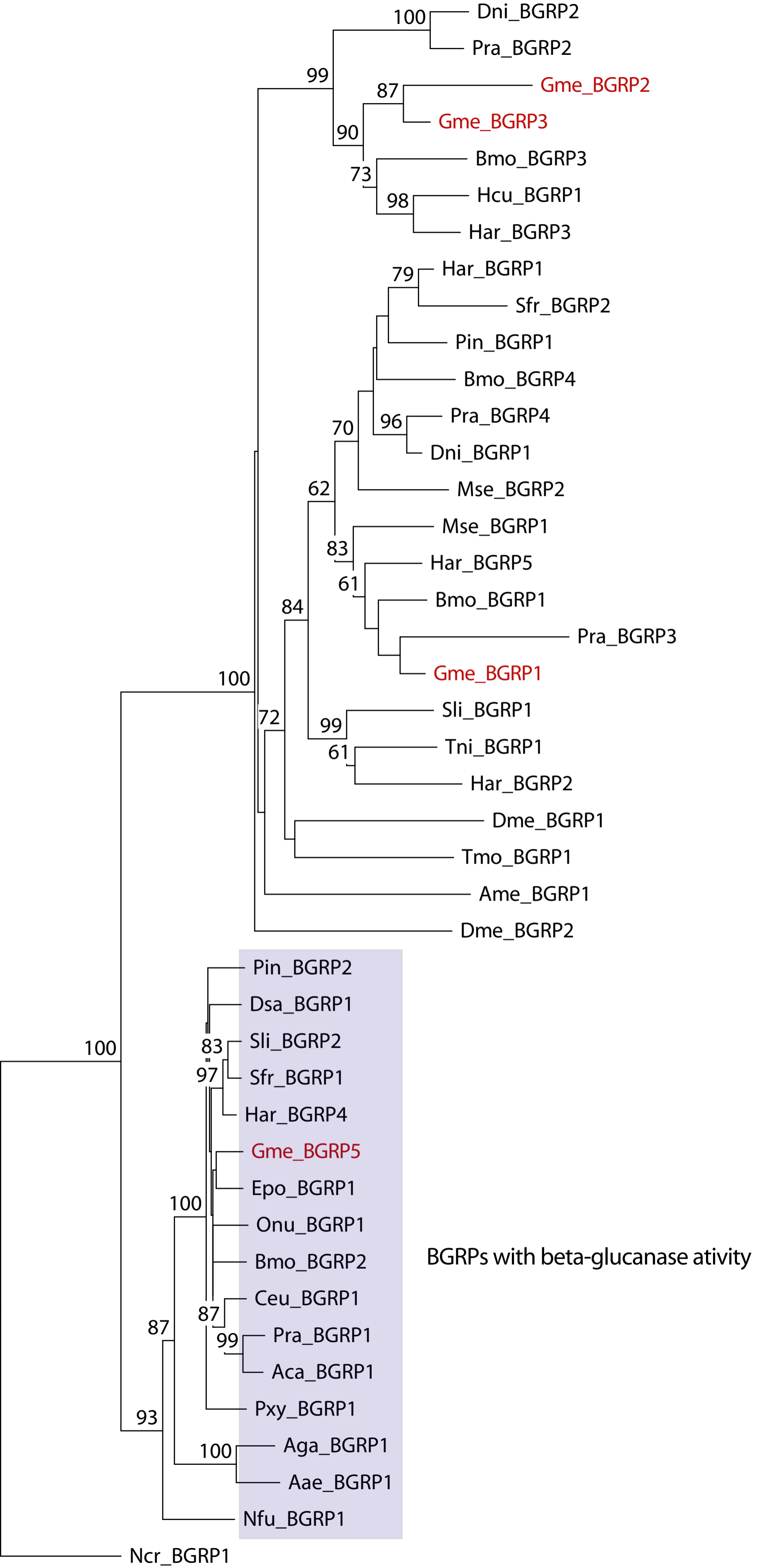

Supplement: Additional file 3 — Gene phylogeny of ßGRP protein sequences. A bayesian phylogenetic tree of insect ßGRP proteins. Bayesian posterior probabilities are shown for all major nodes supported with probability higher than 60%. Amino acid sequence alignments were performed using MAFFT multiple alignment program without the predicted signal peptide and part of the N-terminus as in some cases only partial sequence information was available. Identical residues are boxed with dark shading, and conserved residues are boxed with light shading. All Galleria ßGRP sequences are depicted in red and the group of ßGRP sequences with beta-glucanase activity is shaded. (AdditionalFile-3.pdf) [file 1471-2164-12-308-S3.PDF]

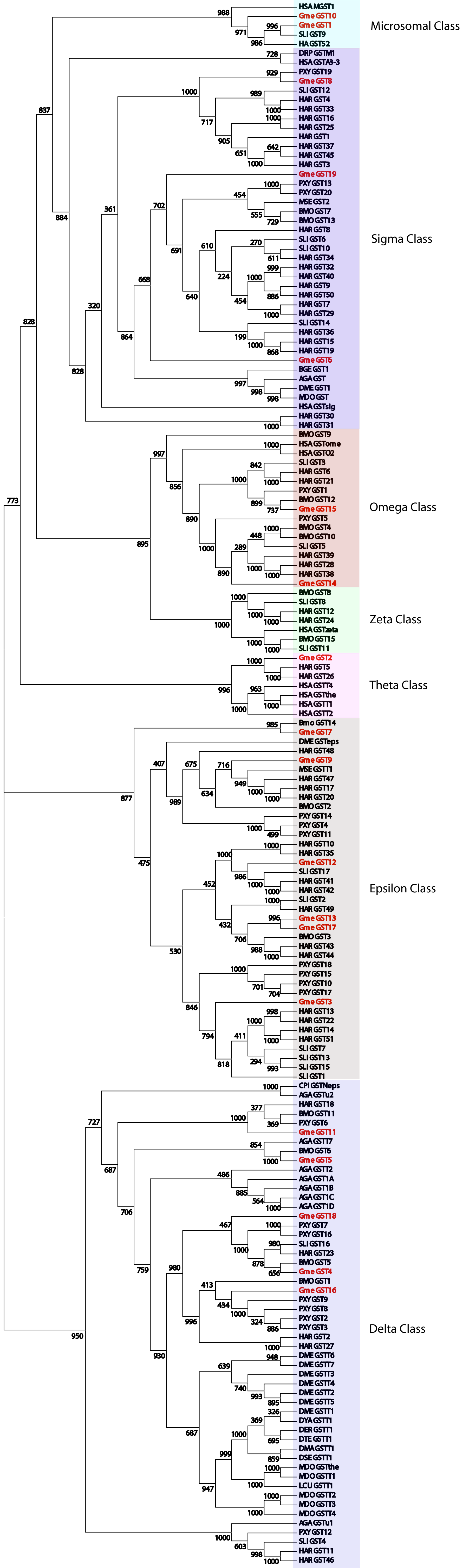

Supplement: Additional file 5 — Gene phylogeny of glutathione S-transferases (GSTs). Neighbour-joining phylogenetic analysis of glutathione-S-transferases from Galleria mellonella (Gme) and other insect species (accession numbers are given). Bootstrap values next to the nodes represent the percentage of 1000 replicate trees that preserved the corresponding clade. Positions containing alignment gaps and missing data were eliminated and not used for the generation of the phylogenetic analysis. An additional Bayesian analysis supported all major nodes with posterior probabilities higher than 60%. [file 1471-2164-12-308-S5.PDF]
