# Supplementary material for: A comprehensive transcriptome and immune-gene repertoire of the lepidopteran model host Galleria mellonella
Source: BMC Genomics. 2011 Jun 11;12:308. doi: 10.1186/1471-2164-12-308 (PMC3224240; doi:10.1186/1471-2164-12-308)
Supplement: Additional file 4 — Amino acid alignments of (A) Spaetzle, (B) Cecropins and (C) cobatoxin sequences from Galleria and other insect species. Deduced from conceptual translation of Galleria transcripts (ESTs) present in the larval dataset. All alignments were performed with MAFFT. Identical residues are boxed with dark shading, and conserved residues are boxed with light shading. [file 1471-2164-12-308-S4.PDF]

A) Spaetzle

```
Mse_Spz-1   RRHSSRLEEPRQRAENGSYKILNNPPK--PCITNRRSQIDSS---NDRVVFPGPTSE---RSYVPEVP-EECKKIGICDS
Bmo_Spz-1   SRSSTKIEEAS-RTENGTFKILNKP----FSIHRRFAQTDDSP--GDRVVFPGLTSRSGVNPYVPEIP-ESCKKMGICDK
Gme_Spz-1   SRRTSTVDEGYRRNSNSSLKIVNDLAKPYPIVSNRRAQEDSKPSLRDSVVFPGPTSR--APLFRPQISDDNCLKHGICES
consensus   .* .....*.  * .*...**.* . .  . ...*..* *...   *.*****.**.     ..*... . *.*.***..

Mse_Spz-1   IPNYPEEHVANIISRLGDKGKVLQIDELDVSDTPDIAQRLGPQEDNMELCSFREKIFYPKAAPDKDGNWFFVVNSKENPV
Bmo_Spz-1   IPNYPREQVEKIVAKLGEK-TEFQFDKLDLPSIPDIAQRAGSFEDSYELCDFRVQIMTPLAGQSDDLKWYHVLNFNENPL
Gme_Spz-1   IPNYPTERVAAILKELENS--KYRFN-VDILDTPEIVQRIGSPDQMMELCNSEEKVVYPEAAYDD--KWYIIINQKKTPR
consensus   ***** * *. *. .*...   .... .*. ..*.*.**.*. .. .*** .......* *. ... .*. ..* ...*.

Mse_Spz-1   QGYKVEICDRQQLPCAEFASFQQG-YEARCIQKYVRRTMLALDPKGQMTDMPLKVPSCCSCVAKLTII
Bmo_Spz-1   QGFRVEICNTTSTGCAKFVTMENN-YNPKCVQKFIFRKMKILSESGEMIERSMKVPSCCSCVATLLG-
Gme_Spz-1   QGFRVEICRPANVPCSSIIYVDESTYNATCIQKHQYRQMAALDESGNLIEKEFKIPSCCSCAISAVNL
consensus   **..****   ...*. ......  *...*.**...* * .*...*..... .*.******..... .
```

B) Cecropin

```
Gme_Cec_C3571        MNFVKVLFFISACILIMLSAVSGAPEPRWKVFKKIERMGQHIRDGIIKAGPAVAVVGQASTIIS--G--
Gme_Cec_C00893       MDFSKIFLFVFAC-VMALTAVSGAPSPKWKIFKKIEKAGRNIRDGIIKAGPAVSVVGEAATIYK--TG-
Gme_Cec_C19824       MKFSKIFFVFFAC-AMFFTTTSAAP---ENFFKEIERAGQRIRDAIISAAPAVETLAQAQKIIK--GGD
Gme_Cec_C20922       -------------------TVSGAPSPKWKFFKKIERVGQNIRDGIIKAGPAVQVVGQAATIYK--GK-
Pxy_Cec_ADA13281     MKLSNIFFFVFMA-FFAVASVSAAP--RWKPFKKLEKVGRNIRDGIIKAGPAVAVIGQATSIARPTGK-
Ara_Cec_AAT94287     MNFGELYFLIFAC-VLALSSVSAAP--KWKIFKKIEHMGQNIRDGLIKAGPAVQVVGQAATIYK--G--
Bmo_Cec_NP001037392  MNFSRALFYVFAV-FLVCASVMAAPEPRWKIFKKIEKVGQNIRDGIIKAGPAVAVVGQAATIAH--GK-
Har_Cec_ADR51147     MNFAKLFFFVFAC-FVVLSTVSGAPEPRWKFFKKVERVGQHIRDGIIKAGPAVAVVGQAAAIAG--K--
Tni_Cec_P50724       MNLVKILFCVFAC---LVFTVTAVPEPRWKFFKKIEKVGQNIRDGIIKAGPAVAVVGQAASITG--K--
Hce_CecA_P01507      MNFSRIFFFVFAC-LTALAMVNAAPEPKWKLFKKIEKVGQNIRDGIIKAGPAVAVVGQATQIAK--G--
Pxu_Cec_ACR82292     MNFGKILFFVMAC-LAALSLTTASP--RWKIFKKIEKVGRNVRDGIIKAGPAVAVVGQAATVVK--G--
consensus            ... .........  . .......* .....**..*..*...**..*.*.***.....*... .  .
```

C) Cobatoxin

```
Gme_Coba_C1521       MKVIILVT-MTVLVLATANALPRIDLYK--LVVSNDIDNSEAMAD-QELSKTAVVSHS--
Gme_Coba_C263        MKVIILVT-MTVLVLATANALPRIDLYK--LVVSNDIDNSEAMADDQELSKTAVVSHS--
Gme_Coba_C997        MKLLLVYV-VAITVLADARILPQINYYKYKLFVPTNIENSIGDAT-EESTNTAVVANN--
Gme_Coba_C17658      MKLLFVCVYLAIIVLTDARILPQMNYYK--LFFPDDIDNSIGDAT-EDSLVAIPTQWSPS
Mse_Coba_ACX49768    MNTKLIIFLATLVILTDA------YVVK-SPKDLSSADSNALEKNSQ---SLLHATYDES
Sfr_Coba_AAQ18900    MK-LILFV-VCAMVMA-AYAVEVISVDD--LLRAYDVSHNKIDGE------SVKMATEA-
Tni_Coba_ABV68851    MKFFIVAV-ISAMVLA-ANA-RFVSLDE--ALFA-DLDRSVDEIK------SIREAKEV-
Har_Coba_ADR51150    MK-LVLVV-ISAIILA-AYA-DVISMDD--LLKAYYLNTNEIGDN------SVQVANQA-
Cno_Coba_O46028      MEGIAKIT-LILLFLF-------VTMHT---FAN----------------------WN--

Gme_Coba_C1521       LYIPMELKGCNNSGCDSVCRALGFNHGRCVSADTCR---------CYN---
Gme_Coba_C263        LYIPMGLRGCNNSGCDSVCRALGFNHGRCVSADTCR---------CYN---
Gme_Coba_C997        LYIPMDLKKCYNNTCDITCKILGYNHGQCVSTSTCH---------CFN---
Gme_Coba_C17658      LHIPMDLKTCYNNTFDIT----GYNHDQCVSSSKINSIKFLFFYITFNK--
Mse_Coba_ACX49768    LYIPMRVSSCSDGICDLGCKILGYPHGRCISANTCQ---------CY----
Sfr_Coba_AAQ18900    LYVPMSLRACTSSACNFVCKFLGYKYGNCVSAETCR---------CYS---
Tni_Coba_ABV68851    LYIPTTIKDCTNPGCDYICKLLGFKHGTCVSSTTCR---------CYS---
Har_Coba_ADR51150    LYVPMNLRSCTSGACNFVCKFLGYKYGTCVSSETCR---------CYS---
Cno_Coba_O46028      --T--EAAVCVYRTCDKDCKRRGYRSGKCIN-NACK---------CYPYGK
```
